# Coding-complete genomes of XBB.1.16, XBB.2.3, FL.4 (alias of XBB.1.9.1.4), and XBB.3 of SARS-CoV-2 Omicron isolated from Bangladesh

**DOI:** 10.1128/MRA.00562-23

**Published:** 2023-09-05

**Authors:** Saikt Rahman, Mohammad Tanbir Habib, Mokibul Hassan Afrad, Mahin Hasan, Anika Tahsin, Mohammad Jubair, Manjur Hossain Khan, Ahmed Nawsher Alam, Emran Kabir Chowdhury, Mustafizur Rahman, Edward Thomas Ryan, Tahmina Shirin, Firdausi Qadri

**Affiliations:** 1 Institute for Developing Science and Health Initiatives, Dhaka, Bangladesh; 2 International Center for Diarrhoeal Disease Research, Dhaka, Bangladesh; 3 Institute of Epidemiology, Disease Control, and Research, Dhaka, Bangladesh; 4 Department of Biochemistry and Molecular Biology, University of Dhaka, Dhaka, Bangladesh; 5 Division of Infectious Diseases, Massachusetts General Hospital, Boston, Massachusetts, USA; 6 Department of Medicine, Harvard Medical School, Boston, Massachusetts, USA; 7 Department of Immunology and Infectious Diseases, Harvard T.H. Chan School of Public Health, Boston, Massachusetts, USA; DOE Joint Genome Institute, Berkeley, California, USA

**Keywords:** XBB, descendants, Omicron sequence, recombinant lineage, XBB.1.16, XBB.2.3, XBB.1.9.1.4, XBB.3, FL.4

## Abstract

We announce the coding-complete genomes of four different strains of SARS-CoV-2 Omicron lineages, XBB.1.16, XBB.2.3, FL.4 (alias of XBB.1.9.1.4), and XBB.3. These strains were obtained between October 2022 and May 2023 from nasopharyngeal swabs of four Bangladeshi individuals, while one of them had a travel history. Genomic data were produced by implementing ARTIC Network-based amplicon sequencing using the Oxford Nanopore Technology.

## ANNOUNCEMENT

On 26 November 2021, the World Health Organization classified severe acute respiratory syndrome coronavirus 2 (SARS-CoV-2) (genus: Betacoronavirus, family: Coronaviridae) Omicron (B.1.1.529) lineages as a variant of concern for having a higher mutation rate and transmission capability (1, 2). The recently evolved Omicron sub-lineage XBB, resulting from the recombination between Omicron lineages BJ.1 and BM.1.1.1, has diversified further to give rise to several descendants that are circulating in Asia and many other parts of the world (3).

As part of routine SARS-CoV-2 detection tests for the nationwide coronavirus disease 2019 (COVID-19) surveillance program [Institute of Epidemiology, Disease Control, and Research (IEDCR) protocol IEDCR/IRB/2020/11], we obtained four SARS-CoV-2-positive samples that were subjected to ONT sequencing. Among these four positive cases, one individual (sample ID: ideSHi-IR-3444) had a travel history from the USA to the Kingdom of Saudi Arabia (24 December 2022) to Bangladesh (3 January 2023) and developed COVID-19 symptoms 3 days after entering Bangladesh. The Novel Coronavirus (2019-nCoV) Nucleic Acid Diagnostic Kit (Sansure Biotech, China) was utilized for real-time reverse transcription-PCR (rRT-PCR) of the viral RNA extracted from nasopharyngeal swab specimen with the QIAamp Viral RNA Minikit (Qiagen, Germany). All four positive cases had a C_T_ value (threshold cycle value) less than 27 for the N gene. Then using the ARTIC Network developed nCoV-2019 sequencing protocol, all these positive samples were subjected to library preparation for whole-genome sequencing (4).

Briefly, the cDNA library was generated from viral RNA utilizing LunaScript RT SuperMix Kit (New England Biolabs, USA), followed by ARTIC v4.1 primer-based multiplex PCR using Q5 high-fidelity DNA polymerase (New England Biolabs, USA) to amplify the genome. dA-tailing of the amplicons was performed utilizing the NEBNext Ultra II end repair/dA-tailing module (New England Biolabs, USA), followed by the ligation to native barcodes EXP-NBD104 (Oxford Nanopore Technologies) through blunt/TA DNA ligase (New England Biolabs, USA). The resultant sequencing libraries were pooled and then sequenced via a FLO-MIN106D flow cell (R9.4.1) for at least 6 hours. MinKNOW v22.12.5 (bionic) was used to accomplish the basecalling and demultiplexing of raw reads. Processed reads were then assembled through the ARTIC guppyplex code script, and the consensus fasta sequences were generated by Medaka v1.4 with ARTIC EPI2ME desktop agent v3.6.2 (FastQ quality control +ARTIC + NextClade v2023.05.17-1833855) (https://artic.network/ncov-2019/ncov2019-bioinformatics-sop.html).

A total of 188,640 reads were obtained for four samples (ranging from 24,270 to 85,554 reads per sample), and a substantially complete genome was obtained for each sample. The genomic information is summarized in Table 1. The lineages of SARS-CoV-2 were assigned in accordance with the proposed nomenclature of Pangolin lineage assignment software v4.3 (https://github.com/cov-lineages/pangolin). Default parameters were used for all tools.

**TABLE 1:** Data for SARS-CoV-2 Omicron lineages

Sample ID	ideSHi-IR-3444	SIRS-3472	SIRS-3473	SIRS-2544
Date of sample collection (yr/mo/date)	2023-01-07	2023-05-15	2023-05-17	2022-10-15
Sampling location	Dhaka	Dhaka	Dhaka	Cox’s Bazar
Age (yr)	70	58	33	20
Gender	Female	Male	Female	Female
Vaccination status	Yes	Yes	Yes	Yes
Vaccination type	Pfizer	Oxford/AstraZeneca	Oxford/AstraZeneca	Sinopharm
Booster dose	Yes (Pfizer)	Yes (Pfizer)	Yes (Moderna)	Yes (Moderna)
Symptoms	Yes	Yes	Yes	Yes
Travel history	Yes	No	No	No
Pangolin lineage	FL.4 (alias of XBB.1.9.1)	XBB.1.16	XBB.2.3	XBB.3
GISAID accession no.	EPI_ISL_16528802	EPI_ISL_17719185	EPI_ISL_17719186	EPI_ISL_17719187
GenBank accession no.	OR098782	OR098783	OR098784	OR098785
SRA accession no.	SRR24848684	SRR24848683	SRR24848682	SRR24848681
No. of reads	85,554	43,561	35,255	24,270
Read depth (×)	~1350.8	~766	~616	~476
Median read length (bp)	468	486	488	493
GC content (%)	37.96	37.93	37.92	37.96

Compared with the Wuhan-Hu-1 strain reference genome (GenBank accession number NC_045512.2), the spike protein-based signature amino acid variations categorized two sequences isolated in May 2023 as sub-lineage XBB.1.16 and XBB.2.3. The ORF1b protein-based signature amino acid variations identified other two sequences as sub-lineage FL.4 (alias of XBB.1.9.1.4) and XBB.3.

The Omicron recombinant lineage XBB is spreading rapidly and evolving to a number of descendants worldwide (3, 5). The detection of co-circulating lineages, rapid sequencing, and data sharing is necessary for fast and informed public health decisions to alleviate COVID-19 consequences.

## Data Availability

The data from this study can be accessed through the GISAID accession numbers listed in Table 1. The NCBI GenBank accession numbers and SRA accession numbers can also be found in Table 1.
